# Predictors of mortality of *Pseudomonas aeruginosa* bacteraemia and the role of infectious diseases consultation and source control; a retrospective cohort study

**DOI:** 10.1007/s15010-024-02326-6

**Published:** 2024-06-20

**Authors:** Matthaios Papadimitriou-Olivgeris, Laurence Senn, Damien Jacot, Benoit Guery

**Affiliations:** 1 Infectious Diseases Service, Lausanne University Hospital, Lausanne, Switzerland; 2 Infection Prevention and Control Unit, Lausanne University Hospital, Lausanne, Switzerland; 3 Infectious Diseases Service, Cantonal Hospital of Sion and Institut Central des Hôpitaux (ICH), Sion, 1951 Switzerland; 4 Institute of Microbiology, Lausanne University Hospital, Lausanne, Switzerland

**Keywords:** *Pseudomonas aeruginosa*, Source control, Infectious diseases consultation, Sepsis, Multidrug resistance

## Abstract

**Purpose:**

To determine predictors of mortality among patients with *Pseudomonas aeruginosa* bacteraemia.

**Methods:**

Retrospective study.

**Setting:**

This study conducted at the Lausanne University Hospital, Switzerland included adult patients with *P. aeruginosa* bacteraemia from 2015 to 2021.

**Results:**

During the study period, 278 episodes of *P. aeruginosa* bacteraemia were included. Twenty (7%) isolates were multidrug-resistant. The most common type of infection was low respiratory tract infection (58 episodes; 21%). Sepsis was present in the majority of episodes (152; 55%). Infectious diseases consultation within 48 h of bacteraemia onset was performed in 203 (73%) episodes. Appropriate antimicrobial treatment was administered within 48 h in 257 (92%) episodes. For most episodes (145; 52%), source control was considered necessary, with 93 (64%) of them undergoing such interventions within 48 h. The 14-day mortality was 15% (42 episodes). The Cox multivariable regression model showed that 14-day mortality was associated with sepsis (*P* 0.002; aHR 6.58, CI 1.95–22.16), and lower respiratory tract infection (*P* < 0.001; aHR 4.63, CI 1.78–12.06). Conversely, interventions performed within 48 h of bacteraemia onset, such as infectious diseases consultation (*P* 0.036; HR 0.51, CI 0.27–0.96), and source control (*P* 0.009; aHR 0.17, CI 0.47–0.64) were associated with improved outcome.

**Conclusion:**

Our findings underscore the pivotal role of early infectious diseases consultation in recommending source control interventions and guiding antimicrobial treatment for patients with *P. aeruginosa* bacteraemia.

**Supplementary Information:**

The online version contains supplementary material available at 10.1007/s15010-024-02326-6.

## Introduction

*Pseudomonas aeruginosa* stands out as a significant cause of bacteraemia, particularly in nosocomial infections, posing a heightened risk to immunocompromised patients, especially those with neutropenia [1–6]. Bacteraemia associated with *P. aeruginosa* carries a notably high mortality rate [1–11]. In fact, among bacteraemias caused by various Gram-negative aerobic bacteria, it exhibits the highest mortality, comparable to that of *Staphylococcus aureus* bacteraemia [1, 4, 5]. 

Several factors common to any pathogen causing bacteraemia are also associated with poorer outcomes among patients with *P. aeruginosa* bacteraemia, such as advanced age, presence of comorbidities, and sepsis [2, 3, 6–8, 10, 11]. A key predictor of mortality is the use of inappropriate empirical antimicrobial treatment, associated to the increased capacity of resistance development of *P. aeruginosa* [2, 6, 10, 11]. Indeed, multidrug-resistant *P. aeruginosa* is associated with increased mortality compared to susceptible strains [6, 9, 10]. Despite this, previously overlooked factors in studies is the timely implementation of source control and the impact of infectious diseases (ID) consultation. While the beneficial roles of the aforementioned interventions have been established for *S. aureus* bacteraemia and candidemia [12–14], only two studies have evaluated the impact of ID consultation in *P. aeruginosa* bacteraemia [8, 11]. Both studies demonstrated its effectiveness, as it influenced both antimicrobial treatment and source control procedures, underscoring its potential for improving patient outcomes [8, 11]. 

Our objective was to determine predictors of mortality in patients with *P. aeruginosa* bacteraemia, specifically by examining the effects of various interventions, including ID consultation, administration of appropriate antimicrobial treatment, and the implementation of source control procedures when deemed necessary.

## Materials and methods

This retrospective study was conducted at Lausanne University Hospital, Switzerland from 2015 to 2021. Inclusion criteria were adult patients (≥ 18 years old) and presence of at least one blood culture for *Pseudomonas aeruginosa*. The sole exclusion criterion was the patient’s refusal of use of clinical data.

BACTEC™ FX BacT/ALERT System (Becton, Dickinson and Company, Franklin Lakes, USAbioMerieux, Marcy l’Etoile, France) was used for incubation of blood cultures. For rapid identification, bacterial pellet preparations were performed on the positive blood cultures (2015 to 2018 based on Croxatto et al. method, and from 2018 with the rapid BACpro® II Nittobo Medical Co., Tokyo, Japan) [15]. Bacterial pellets were identified with a Matrix-assisted laser desorption-ionization time of flight mass spectrometry (MALDI-TOF MS; Bruker Daltonics, Bremen, Germany) was used for species identification from 07:00 to 19:00. Susceptibility results (Vitek® 2, bioMerieux Marc l’Etoile, France and Kirby-Bauer methods) were obtained from the microbiology laboratory database and assessed in accordance with the EUCAST criteria [16]. 

Fourteen-day all-cause mortality was the primary outcome. Data on demographics (age, sex), comorbidities, Charlson Comorbidity Index, ID consultation, antimicrobial treatment, source control, the presence of sepsis, and the site of infection were retrieved from patients’ electronic health records. All data were collected, stored and managed using REDCap by an ID specialist. REDCap electronic data capture tools are hosted at Lausanne University Hospital. REDCap (Research Electronic Data Capture) is a secure, web-based software platform designed to support data capture for research studies [17, 18]. 

In our institution, ID consultants receive notification regarding patients with positive blood cultures following species identification. For *P. aeruginosa* bacteraemia, ID consultation is not mandatory. However, patients undergoing induction or consolidation/maintenance chemotherapy, those with lymphoma or multiple myeloma undergoing autologous hematopoietic stem cell transplantation are hospitalized in the ID service while those after allogeneic stem cell or solid organ transplantation are closely monitored by ID consultants. The decision to perform follow-up blood cultures is at the discretion of the treating physician.

The date of collection of the first positive blood culture was defined as bacteraemia onset. A new episode was included if more than 30 days had elapsed since the cessation of antibiotic treatment for the initial bacteraemia. The Sepsis-3 International Consensus criteria were used for sepsis definition [19]. The determination of the infection site was based on the assessment by the ID consultant, or treating physician, taking into account clinical, radiological, microbiological, and operative findings. Appropriate antimicrobial treatment was defined as the initiation of at least one antimicrobial agent with in vitro activity against the infecting isolate. Source control was deemed necessary in the following situations: removal of venous catheter in patients with catheter-related bacteraemia or bacteraemia of unknown origin in the presence of a venous catheter; imaging-guided or surgical drainage of infected collections, such as abscesses, empyema, etc.; joint fluid drainage; cardiac surgery in endocarditis patients when indicated for heart failure; and correction of urinary-tract obstruction. We used the cutoff of 48 h to define early interventions (antimicrobial treatment initiation, source control, ID consultation) from bacteraemia onset Multidrug-resistance was defined as resistance to at least one agent in three or more antimicrobial categories [20]. Time to positivity of blood cultures was evaluated in episodes where at least one bottle grew only *P. aeruginosa*.

SPSS version 26.0 (SPSS, Chicago, IL, USA) were used for data analyses. Categorical variables were analyzed using the *chi*-square or Fisher exact test and continuous variables with Mann–Whitney *U* test. Two univariate logistic regression models were assessed with 14-day and 30-day mortality as dependent variables. Clinically relevant non collinear covariates, assessed through variance inflation factor, were used in multivariable analysis. After checking Cox assumptions, two multivariable Cox proportional hazards regression models were performed with 14-day and 30-day mortality as the time-to-event. Adjusted Hazzard ratios (aHRs) and 95% confidence intervals (CIs) were calculated to evaluate the strength of any association. All statistic tests were 2-tailed and *P* < 0.05 was considered statistically significant. We finally performed Kaplan-Meier curves of the survival probability of patients with *P. aeruginosa* bacteraemia according to appropriate source control within 48 h from bacteraemia onset.

## Results

**Fig. 1:**
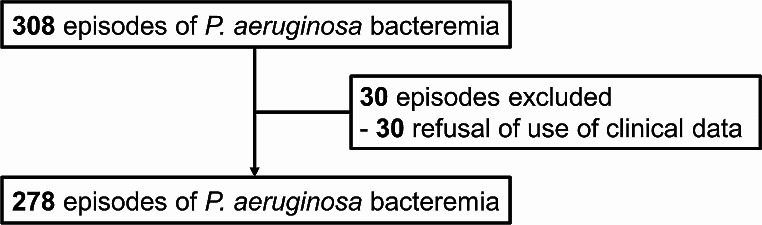
Flowchart of patients’ inclusion

Among the 308 episodes of *P. aeruginosa* bacteraemia, 278 episodes were included, involving 261 patients (Fig. 1). Twelve and five patients had two and three episodes, respectively. These 22 subsequent episodes of bacteremia, occurred at a median of 2 months from the previous episode (interquartile range: 2–3 months). Resistance was observed in 36 (13%) isolates for piperacillin/tazobactam, 37 (13%) for ceftazidime, 28 (10%) for cefepime, 39 (14%) for imipenem, 23 (8%) for meropenem, 9 (3%) for amikacin, and 31 (11%) for ciprofloxacin. Twenty (7%) isolates were classified as multidrug-resistant. Resistance to colistin was observed in 5% (one isolate among 22 tested), to ceftazidime/avibactam in 40% (6 out of 15), and to ceftolozane/tazobactam in 29% (5 out of 17). The susceptibility profiles and the respective administered antimicrobial treatment of the 20 episodes due to multidrug-resistant *P. aeruginosa* isolates are shown in Supplementary Table 1. Follow-up blood cultures were conducted until sterilization in 221 (80%) episodes, among which 28 (13%) exhibited persistent bacteraemia for at least 48 h.

The most common type of infection was low respiratory tract infection (58 episodes; 21%), followed by catheter-related (54; 19%) and abdominal infection (54; 19%). Sepsis was present in the majority of episodes (152; 55%).

ID consultation was performed in 224 (81%) episodes, with 203 (73%) consultations provided within 48 h of bacteraemia onset. Appropriate antimicrobial treatment was administered within 48 h of bacteraemia onset in 257 (92%) episodes. The impact of ID consultation on antimicrobial treatment is shown in Supplementary Table 2. Among the 80 episodes where appropriate antimicrobial treatment was not administered within 24 h of bacteraemia onset, 49 (61%) had an early ID consultation. More episodes in the early ID consultation group had appropriate antimicrobial treatment initiated within 48 h compared to those without ID consultation in the same timeframe (90% *versus* 52%; *P* < 0.001). Among the 60 episodes where appropriate treatment by carbapenems was administered within the first 24 h, 48 (80%) had early ID consultation. More episodes in the early ID consultation group had a de-escalation to ceftazidime, cefepime, or piperacillin/tazobactam within 48 h compared to those without ID consultation in the same timeframe (48% *versus* 8%; *P* < 0.001). For the majority of episodes (145; 52%), source control was considered necessary, with 93 (64%) of them undergoing such interventions within 48 h.

The overall 14-day mortality was 15% (42 episodes), while 30-day mortality was 22% (60 episodes). Table 1 shows the comparison of survivors and not survivors at day 14. Sepsis was more prevalent among deceased patients (93% *versus* 48%; *P* < 0.001). Lower respiratory tract infections were more common among deceased patients (60% *versus* 14%; *P* < 0.001). Early ID consultation (50% *versus* 77%; *P* 0.001) and source control (25% versus 68%; *P* < 0.001) were less frequently observed in deceased patients. No association was found between multidrug-resistance, time to blood culture positivity, appropriate antimicrobial treatment within 48 h, and 14-day mortality.

**Table 1:** Comparison of survivors and not survivors at day 14

	Survivors (*n* = 236)		Non-survivors (*n* = 42)			*P*
Demographics						
Male sex	168	71%		24	57%	0.102
Age (years)	65	54–75		74	64–79	0.003
Age > 60 years	141	60%		34	81%	0.009
Co-morbidities						
Malignancy (solid organ or haematologic)	91	39%		19	35%	0.494
Immunosuppression^a^	93	39%		14	33%	0.496
Diabetes mellitus	52	22%		14	33%	0.119
Chronic kidney disease (moderate or severe)	53	23%		7	17%	0.542
Obesity (body mass index ≥ 30 kg/m^2^)	36	15%		6	14%	1.000
Chronic obstructive pulmonary disease	23	10%		5	12%	0.589
Congestive heart failure	12	5%		2	5%	1.000
Cirrhosis	9	4%		2	5%	0.675
Charlson Comorbidity Index	5	3–7		6	4–8	0.028
Charlson Comorbidity Index > 4	118	50%		29	69%	0.029
Setting of bacteraemia onset						
Community	28	12%		7	17%	
Healthcare-associated	45	19%		5	12%	
Nosocomial	163	69%		30	71%	0.857
Microbiological data						
Two or more blood cultures positive (initial blood cultures)	142	60%		33	55%	0.609
Multidrug-resistance	19	8%		1	2%	0.328
Persistent bacteraemia (≥ 48 h)	25	11%		3	7%	0.780
Polymicrobial bacteraemia	53	23%		7	17%	0.542
Time to positivity (hours)^b^	15	12–19		15	11–17	0.257
Type of infection						
Lower respiratory tract infection	33	14%		25	60%	< 0.001
Catheter-related	51	22%		3	7%	0.033
Abdominal infection	51	22%		3	7%	0.033
Urinary tract infection	36	15%		5	12%	0.813
Unknown origin	32	14%		3	7%	0.319
Other foci	35	15%		4	10%	0.473
Sepsis	113	48%		39	93%	< 0.001
Management						
Infectious diseases consultation	202	86%		22	52%	< 0.001
Infectious diseases consultation within 48 h	182	77%		21	50%	0.001
Source control						
Not warranted	103	44%		30	71%	
Warranted and performed within 48 h	90	38%		3	7%	
Warranted, but not performed within 48 h	43	18%		9	21%	< 0.001
Antimicrobial initiation within 48 h	232	98%		41	98%	0.562
Appropriate antimicrobial within 48 h	220	93%		37	88%	0.336

Data are depicted as number and percentage or median and Q1-3

^a^ongoing immunosuppressive treatment at bacteraemia onset, intravenous chemotherapy in the 30 days prior to bacteraemia onset, AIDS, neutropenia and asplenia

^b^evaluated in 268 episodes where at least one bottle grew only *P. aeruginosa*

The Cox multivariable regression model of 14-day mortality is presented in Table 2. Fourteen-day mortality was associated with sepsis (*P* 0.002; aHR 6.58, CI 1.95–22.16), and lower respiratory tract infection (*P* < 0.001; aHR 4.63, CI 1.78–12.06). Conversely, interventions performed within 48 h of bacteraemia onset, such as ID consultation (*P* 0.036; HR 0.51, CI 0.27–0.96), and source control (*P* 0.009; aHR 0.17, CI 0.47–0.64) were associated with improved outcome.

**Table 2:** Univariable and multivariable Cox proportional hazard regression of 14-day mortality among patients with bacteraemia due to *P. aeruginosa*

	Univariable analysis		Multivariable Cox regression	
	*P*	HR (95% CI)	*P*	aHR (95% CI)
Charlson Comorbidity Index > 4	0.025	2.11 (1.10–4.06)	0.051	1.96 (0.99–3.84)
Sepsis	< 0.001	12.33 (3.81–39.92)	0.002	6.58 (1.95–22.16)
Lower respiratory tract infection	< 0.001	7.22 (3.89–13.40)	0.002	4.63 (1.78–12.06)
Infectious diseases consultation within 48 h	< 0.001	0.31 (017-0.57)	0.036	0.51 (0.27–0.96)
Source control				
Warranted, but not performed within 48 h	reference		reference	
Warranted and performed within 48 h	0.010	0.18 (0.05–0.67)	0.009	0.17 (0.47–0.64)
Not warranted	0.353	1.42 (0.68-3.00)	0.124	0.47 (0.15–1.26)

CI: confidence interval; aHR: adjusted hazard ratio

**Fig. 2:**
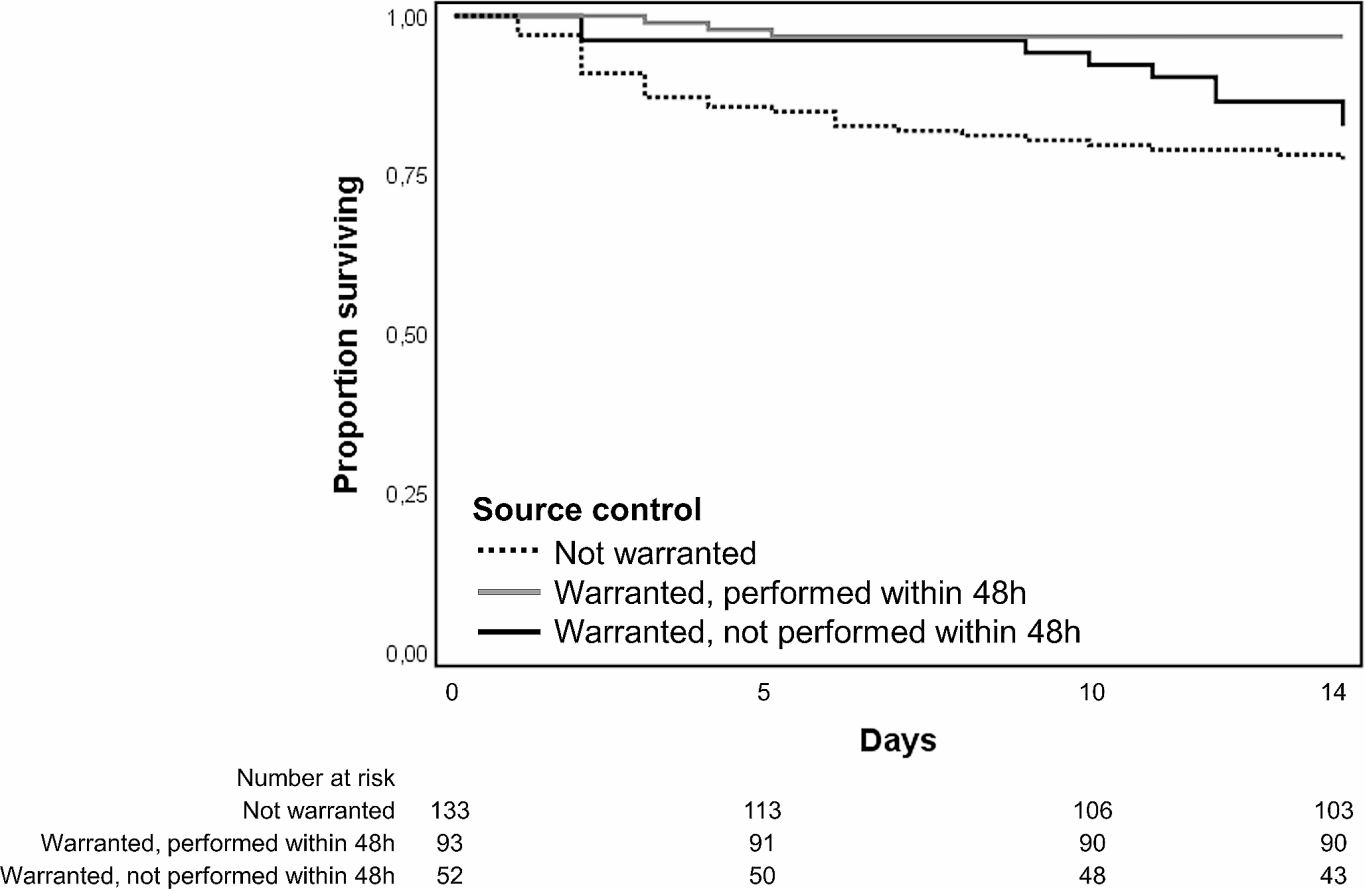
Kaplan–Meier curves of the survival probability curves for episodes with *P. aeruginosa* bacteraemia categorized by the requirement for and execution of source control within 48 h. Comparison of episodes where source control was performed *versus* those where it was indicated but not performed: Log-rank test, *P* 0.004. Comparison of episodes where source control was performed versus those where source control was not warranted: Log-rank test, *P* < 0.001. Comparison of episodes where source control was not performed versus those where source control was not warranted: Log-rank test, *P* 0.267

Figure 2 illustrates Kaplan–Meier survival probability curves for episodes with *P. aeruginosa* bacteraemia categorized by the requirement for and execution of source control within 48 h. Source control within 48 h was linked to a more favorable outcome when compared to episodes where it was warranted but not performed (Log-rank test, *P* 0.004).

The comparison of survivors and not survivors at day 30 and the Cox multivariable regression model of 30-day mortality are presented in Supplementary Tables 3 and 4, respectively. Thirty-day mortality was associated with a Charlson comorbidity index superior to 4 (*P* 0.001; aHR 2.83, CI 1.58–5.09), sepsis (*P* 0.002; aHR 7.48, CI 1.52–6.84), and lower respiratory tract infection (*P* < 0.001; aHR 4.60, CI 2.15–9.81). Conversely, interventions performed within 48 h of bacteraemia onset, such as ID consultation (*P* 0.049; HR 0.59, CI 0.35–0.99), and source control (*P* 0.009; aHR 0.29, CI 0.11–0.74) were associated with improved outcome.

## Discussion

In this study, we investigated predictors of mortality among patients with *P. aeruginosa* bacteraemia and underscored the importance of ID consultation and timely source control in enhancing outcomes.

The mortality rate in our cohort (22%) was similar to previous research [3–5, 8, 11], but lower than studies conducted in regions with higher resistance rates [6, 7, 9, 10]. The relatively low incidence of multidrug resistance among *P. aeruginosa* isolates, coupled with close monitoring by ID specialists for high-risk patients (such as those undergoing chemotherapy or transplantation), likely contributed to the higher rate of appropriate antimicrobial treatment within the first 48 h of bacteraemia onset in the present study, compared to prior research [2, 3, 6, 7, 10, 11]. 

The most important finding was the positive impact of ID consultation on managing *P. aeruginosa* infections, particularly in guiding antimicrobial therapy and source control measures. Consistent with two previous studies on *P. aeruginosa* bacteraemia, our findings emphasize the importance of ID consultation in improving patient outcomes [8, 11]. The aforementioned studies suffered from survival bias, with only patients surviving long enough receiving ID consultation. One of these studies did not include a timeframe for the realization of ID consultation [8], while the other included ID consultation within two weeks from the onset of bacteraemia [11]. This issue was addressed in our study by implementing a timeframe of 48 h from the onset of bacteraemia. In the present study, the early involvement of ID consultants led to an earlier initiation of appropriate treatment and earlier de-escalation from carbapenems to other beta-lactams with a narrower spectrum. Although the dosages of the administered antimicrobials were not collected in the present study, a previous study from our institution showed that ID consultation among all *P. aeruginosa* infections led to an increase in the administered dosages of non-carbapenem antibiotics [21]. However, the influence of ID consultation might have been underestimated in our study, as patients at higher risk of mortality, such as those with neutropenia following chemotherapy for hematologic malignancies or undergoing transplantation, were either closely monitored by ID consultants or hospitalized in the ID service, thus *de facto* receiving consultation within 48 h [3]. 

In a prior study involving patients with infections due to multidrug-resistant pathogens, ID consultation proved beneficial for methicillin-resistant *S. aureus* and multidrug-resistant Enterobacteriaceae, but not for *P. aeruginosa*, likely due to the limited number of patients included with infection caused by this pathogen [22]. Furthermore, although previous research extensively evaluated the effect of appropriate empirical antimicrobial treatment on survival in *P. aeruginosa* bacteraemia, the role of early source control measures among *P. aeruginosa* bacteraemic patients was rarely investigated [8]. The impact of timely source control on better outcomes (survival, clearance of bacteraemia or candidaemia) has been demonstrated in various types of infections, such as intra-abdominal infections, necrotizing fasciitis, sepsis, and bloodstream infections caused by different pathogens, including *S. aureus*, streptococci, and *Candida* spp [13, 14, 23–26]. Especially in sepsis which was associated with poorer outcomes in our study, prompt source control is recommended by the Surviving Sepsis Campaign Guidelines to enhance management and improve outcomes [27]. This recommendation is supported by a study on critically ill patients, regardless of the causative pathogen [28]. 

As previously demonstrated, an elevated Charlson comorbidity index, which encompasses age and various comorbid conditions, was correlated with increased 30-day mortality [1–3, 7, 8, 10, 11]. Previous research has highlighted the significance of the focus of infection in determining outcomes, with bacteraemias secondary to lower respiratory tract or pulmonary infections associated with worse prognoses [1, 2, 29, 30]. 

In prior studies, the time to positivity of blood cultures, serving as an indirect measure of the microbial load of the infecting organism, was found to be indicative of poorer outcomes among patients with *P. aeruginosa* bacteraemia [29, 30]. No such correlation was identified in the current study, underscoring the limitations in evaluating the time to positivity of blood cultures. Variability in protocols for blood culture drawing, processing, and incubation across different centers can lead to discrepancies in the time to positivity results.

The current study is subject to several limitations. Firstly, it was a retrospective analysis conducted at a single center in a setting with low resistance rates, thus caution must be exercised when generalizing our findings, particularly to settings with higher resistance rates. Moreover, factors such as decisions to restrict treatment and the readiness of surgeons or interventional radiologists to undertake source control procedures could influence the relationship between outcome and prompt interventions. In our study, among episodes warranting source control intervention, only three patients succumbed or had care withdrawn within the 48-hour timeframe, minimizing their impact on our results.

In conclusion, we have demonstrated the beneficial role of timely ID consultation in patients with *P. aeruginosa* bacteraemia, aligning it with other pathogens such as *S. aureus*, *Candida* spp., enterococci, and streptococci, thereby advocating for ID consultation as an integral component of management to enhance patient outcomes. Additionally, we have underscored the importance of implementing source control interventions when appropriate. Future studies are needed to evaluate the impact of comprehensive approach with early interventions such as ID consultation, tailored antimicrobial treatment, and source control in enhancing patient outcomes.

## Electronic supplementary material

Below is the link to the electronic supplementary material.

Supplementary Material 1

## Data Availability

The data that support the findings of this study are available from the corresponding author upon reasonable request.

## Declarations

### Statements and Declarations

A part of this work was presented at the 34th European Congress of Clinical Microbiology and Infectious Diseases on 27–30 April 2024 in Barcelona, Spain.
