# Supplementary material for: Predictors of mortality of Pseudomonas aeruginosa bacteraemia and the role of infectious diseases consultation and source control; a retrospective cohort study
Source: Infection. 2024 Jun 20;53(1):117–24. doi: 10.1007/s15010-024-02326-6 (PMC11825531; doi:10.1007/s15010-024-02326-6)
Supplement: Supplementary file 1 — Supplementary Material 1 [file 15010_2024_2326_MOESM1_ESM.pdf]

Supplementary Table 1. Susceptibility profile and antimicrobial treatment of the 20 bacteraemia episodes due to multidrug-resistant *P. aeruginosa*

Episode	Susceptibility profile							Antimicrobial treatment	
	**PIT**	**CTZ**	**CEP**	**IMI**	**MER**	**AMI**	**CIP**	**Empiric**	**Targeted**
1	**R**	**R**	**R**	**R**	**R**	S	**R**	Colistin	Ceftolozane/tazobactam
2	**R**	**R**	**R**	**R**	**R**	S	**R**	Meropenem, amikacin	Cefiderocol
3	**R**	**R**	**R**	**R**	**R**	S	**R**	Ceftazidime/avibactam, amikacin	Ceftazidime/avibactam
4	**R**	**R**	**R**	**R**	**R**	S	**R**	Piperacillin/tazobactam	Ceftazidime/avibactam
5	**R**	**R**	**R**	**R**	**R**	S	**R**	Piperacillin/tazobactam	Ceftolozane/tazobactam
6	**R**	**R**	**R**	**R**	**R**	S	**R**	Colistin, amikacin	Colistin
7	**R**	**R**	**R**	**R**	**R**	S	**R**	Ceftolozane/tazobactam, amikacin	Ceftolozane/tazobactam
8	**R**	**R**	**R**	**R**	**R**	**R**	**R**	Meropenem	Colistin
9	**R**	**R**	**R**	**R**	**R**	**R**	**R**	Meropenem	Colistin
10	**R**	**R**	**R**	**R**	**R**	**R**	**R**	Ceftazidime/avibactam, amikacin	Ceftazidime/avibactam
11	**R**	**R**	**R**	**R**	**R**	**R**	**R**	Cefepime	Colsitin
12	**R**	**R**	**R**	**R**	**R**	**R**	**R**	Cefepime	Colsitin
13	**R**	**R**	**R**	**R**	**R**	**R**	**R**	Piperacillin/tazobactam, amikacin	Colsitin
14	**R**	**R**	**R**	**R**	S	S	**R**	Piperacillin/tazobactam	Meropenem
15	**R**	**R**	**R**	**R**	**R**	**R**	S	Ceftazidime/avibactam, colistin	Ceftazidime/avibactam
16	**R**	**R**	**R**	**R**	**R**	S	S	Meropenem, amikacin	Ciprofloxacin
17	**R**	**R**	**R**	**R**	S	S	S	Imipenem, ciprofloxacin	Meropenem
18	**R**	**R**	S	**R**	**R**	**R**	**R**	Piperacillin/tazobactam	Cefepime
19	**R**	**R**	S	**R**	**R**	S	S	Ceftolozane/tazobactam	Ceftolozane/tazobactam
20	**R**	S	S	**R**	S	S	**R**	Meropenem, amikacin	Meropenem

AMI: amikacin; CEP: cefepime; CIP: ciprofloxacin; CTZ: ceftazidime; IMI: imipenem; MER: meropenem; PIT: piperacillin/tazobactam; R: resistant; S: susceptible

Supplementary Table 2. Impact of early infectious diseases consultation on antimicrobial treatment administration

	**No early ID consultation**		**Early ID consultation**		***P***
No appropriate antimicrobial treatment within 24h[a]	**n=31**		**n=49**		
Initiation of appropriate antimicrobial within 48h	15	48%	5	10%	<0.001
Initiation of appropriate antimicrobial after 48h	16	52%	44	90%	
Appropriate antimicrobial treatment within 24h[b]	**n=44**		**n=154**		
Escalation	3	7%	14	9%	0.768
Carbapenem within 24h (appropriate treatment)	**n=12**		**n=48**		
Continuation of carbapenem	11	92%	25	52%	0.019
De-escalation to ceftazidime, cefepime or piperacillin/tazobactam	1	8%	23	48%	
Combination treatment within 24h	**n=9**		**n=43**		
Continuation of combination treatment	1	11%	12	28%	0.420
Cessation of aminoglycoside or quinolones	8	89%	31	72%	

Data are depicted as number and percentage

[a]no antimicrobial treatment: 12 episodes; administration of antimicrobial treatment without antipseudomonal activity: 45 episodes; administration of antimicrobial treatment with antipseudomonal activity, but *in-vitro* resistance: 23

[b]piperacillin/tazobactam: 79 episodes; carbapenem: 60 episodes; ceftazidime or cefepime: 47 episodes; colistin 7 episodes; ceftolozane/tazobactam or ceftazidime/avibactam: 5 episodes; combination treatment with either aminoglycosides or quinolones: 52 episodes

Supplementary Table 3. Comparison of survivors and not survivors at day 30

	**Survivors (n=218)**		**Non-survivors (n=60)**		***P***
Demographics					
Male sex	155	71%	37	72%	0.207
Age (years)	63	52-75	74	64-79	<0.001
Age >60 years	126	58%	49	82%	0.001
Co-morbidities					
Malignancy (solid organ or haematologic)	80	37%	30	50%	0.074
Immunosuppression[a]	83	38%	24	40%	0.881
Diabetes mellitus	48	22%	18	30%	0.230
Chronic kidney disease (moderate or severe)	47	22%	13	22%	1.000
Obesity (body mass index ≥30 kg/m^2)	34	16%	8	13%	0.839
Chronic obstructive pulmonary disease	22	10%	6	10%	1.000
Congestive heart failure	21	6%	2	3%	0.741
Cirrhosis	7	3%	4	7%	0.259
Charlson Comorbidity Index	4	2-7	6	4-8	<0.001
Charlson Comorbidity Index >4	103	47%	44	73%	<0.001
Setting of bacteraemia onset					
Community	25	12%	10	17%	
Healthcare-associated	41	19%	9	15%	
Nosocomial	152	70%	41	68%	0.875
Microbiological data					
Two or more blood cultures positive (initial blood cultures)	132	61%	33	55%	0.461
Multidrug-resistance	17	8%	3	5%	0.581
Persistent bacteraemia (≥48 hours)	25	12%	4	7%	0.347
Polymicrobial bacteraemia	48	22%	12	20%	0.860
Time to positivity (hours)[b]	15	12-19	14	12-17	0.202
Type of infection					
Lower respiratory tract infection	26	12%	32	53%	<0.001
Catheter-related	50	23%	4	7%	0.003
Abdominal infection	47	22%	7	12%	0.099
Urinary tract infection	35	16%	6	10%	0.306
Unknown origin	31	14%	4	7%	0.130

Other foci	32	15%	7	12%	0.677
Sepsis	101	46%	51	85%	<0.001
Management					
Infectious diseases consultation	189	87%	35	58%	<0.001
Infectious diseases consultation within 48 hours	170	78%	33	55%	0.001
Source control					
Not warranted	92	42%	41	68%	
Warranted and performed within 48 hours	86	39%	7	12%	
Warranted, but not performed within 48 hours	40	18%	12	20%	<0.001
Antimicrobial initiation within 48 hours	214	98%	59	98%	1.000
Appropriate antimicrobial within 48 hours	203	93%	54	90%	0.414

Data are depicted as number and percentage or median and Q1-3

[a]ongoing immunosuppressive treatment at bacteraemia onset, intravenous chemotherapy in the 30 days prior to bacteraemia onset, AIDS, neutropenia and asplenia.

[b]evaluated in 268 episodes where at least one bottle grew only *P. aeruginosa*

Supplementary Table 4. Univariable and multivariable Cox proportional hazard regression of 30-day mortality among patients with bacteraemia due to *P. aeruginosa*

	Univariable analysis		Multivariable Cox regression	
	P	**HR (95% CI)**	***P***	**aHR (95% CI)**
Charlson Comorbidity Index >4	0.001	2.68 (1.51-4.75)	0.001	2.83 (1.58-5.09)
Sepsis	<0.001	5.69 (2.80-11.57)	0.002	3.22 (1.52-6.84)
Lower respiratory tract infection	<0.001	6.17 (3.70-10.27)	<0.001	4.60 (2.15-9.81)
Infectious diseases consultation within 48 hours	<0.001	0.38 (0.23-0.63)	0.049	0.59 (0.35-0.99)
Source control				
Warranted, but not performed within 48 hours	reference		reference	
Warranted and performed within 48 hours	0.012	0.30 (0.12-0.77)	0.009	0.29 (0.11-0.74)
Not warranted	0.253	1.46 (0.77-2.77)	0.160	0.54 (0.23-1.28)

CI: confidence interval; aHR: adjusted hazard ratio
